# 4-(5-Chloro­thio­phen-2-yl)-1,2,3-selenadiazole

**DOI:** 10.1107/S1600536812049549

**Published:** 2012-12-12

**Authors:** Paramasivam Sugumar, Subramaniyan Sankari, Paramasivam Manisankar, Mondikalipudur Nanjappa Gounder Ponnuswamy

**Affiliations:** a Centre of Advanced Study in Crystallography and Biophysics, University of Madras, Guindy Campus, Chennai 600 025, India; b Department of Chemistry, Sri Sarada College for Women (Autonomus), Fairlands, Salem 636 016, India; c Department of Industrial Chemistry, Alagappa University, Karaikudi 630 003, India

## Abstract

In the title compound, C_6_H_3_ClN_2_SSe, the selenadiazole and chloro­thio­phene rings are almost coplanar [dihedral angle = 5.24 (15)°]. In the crystal, C—H⋯N inter­actions link the mol­ecules into chains extending along the *b*-axis direction. C—H⋯π inter­actions also occur.

## Related literature

For the biological activity of selenadiazole derivatives, see: El-Bahaie *et al.* (1990 ▶); El-Kashef *et al.* (1986 ▶); Padmavathi *et al.* (2002 ▶); Plano *et al.* (2010 ▶); Stadtman (1991 ▶); Velusamy *et al.* (2005 ▶). For bond-length data, see: Allen *et al.* (1987 ▶).
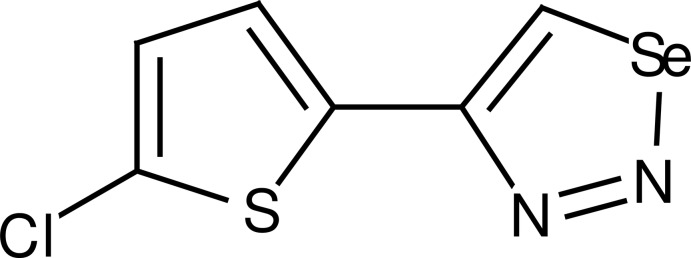


## Experimental

### 

#### Crystal data

C_6_H_3_ClN_2_SSe
*M* *_r_* = 249.57
Monoclinic, 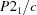

*a* = 6.0412 (3) Å
*b* = 19.5870 (11) Å
*c* = 7.2010 (4) Å
β = 110.257 (3)°
*V* = 799.38 (7) Å^3^
*Z* = 4
Mo *K*α radiation
μ = 5.22 mm^−1^
*T* = 293 K
0.22 × 0.20 × 0.18 mm

#### Data collection

Bruker SMART APEX CCD detector diffractometer
Absorption correction: multi-scan (*SADABS*; Bruker, 2008 ▶) *T*_min_ = 0.330, *T*_max_ = 0.391
7064 measured reflections
1978 independent reflections
1558 reflections with *I* > 2σ(*I*)
*R*_int_ = 0.044

#### Refinement

*R*[*F* ^2^ > 2σ(*F* ^2^)] = 0.039
*wR*(*F* ^2^) = 0.121
*S* = 1.01
1978 reflections
100 parameters
H-atom parameters constrained
Δρ_max_ = 0.44 e Å^−3^
Δρ_min_ = −0.52 e Å^−3^

### 

Data collection: *APEX2* (Bruker, 2008 ▶); cell refinement: *SAINT* (Bruker, 2008 ▶); data reduction: *SAINT*; program(s) used to solve structure: *SHELXS97* (Sheldrick, 2008 ▶); program(s) used to refine structure: *SHELXL97* (Sheldrick, 2008 ▶); molecular graphics: *ORTEP-3* (Farrugia, 2012 ▶); software used to prepare material for publication: *SHELXL97* and *PLATON* (Spek, 2009 ▶).

## Supplementary Material

Crystal structure: contains datablock(s) global, I. DOI: 10.1107/S1600536812049549/bt6854sup1.cif

Structure factors: contains datablock(s) I. DOI: 10.1107/S1600536812049549/bt6854Isup2.hkl

Supplementary material file. DOI: 10.1107/S1600536812049549/bt6854Isup3.cml

Additional supplementary materials:  crystallographic information; 3D view; checkCIF report

## Figures and Tables

**Table 1:** Hydrogen-bond geometry (Å, °)

*D*—H⋯*A*	*D*—H	H⋯*A*	*D*⋯*A*	*D*—H⋯*A*
C1—H1⋯N1^i^	0.93	2.62	3.545 (5)	171

Symmetry code: (i) 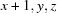.

**Fig. 1.**
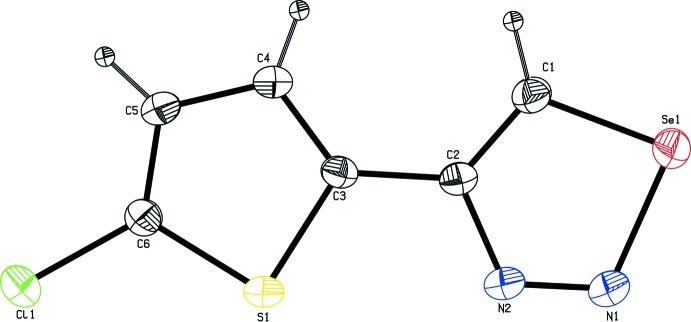
The molecular structure of the title compound, showing the atomic numbering and displacement ellipsoids drawn at 30% probability level.

**Fig. 2.**
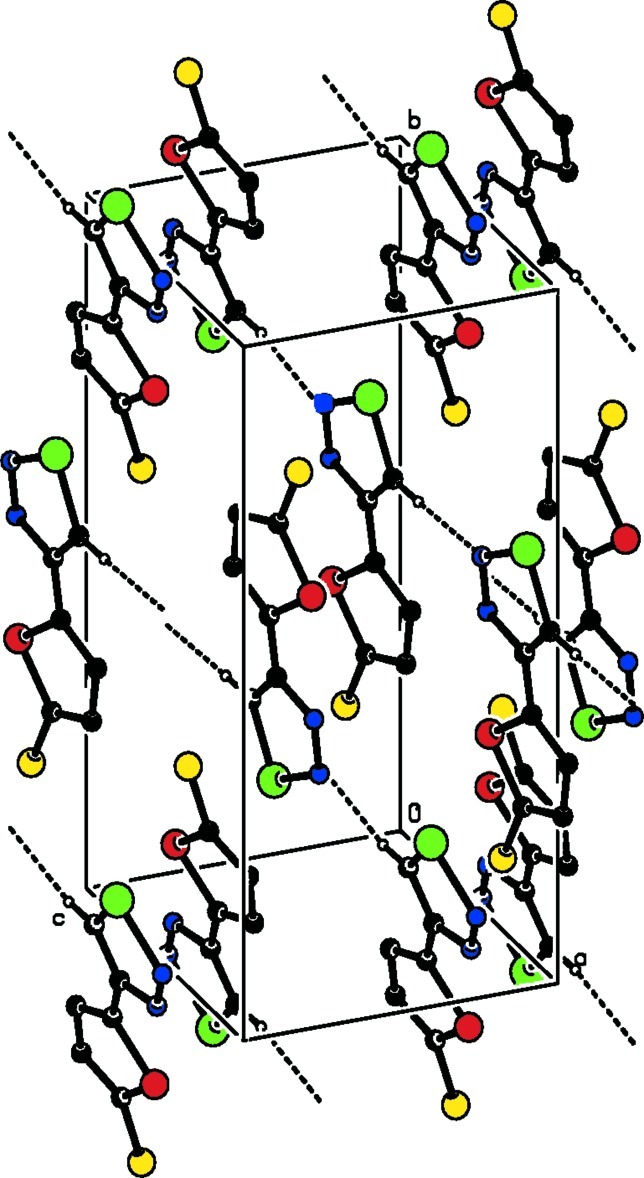
The crystal packing.

## supplementary crystallographic information

### Comment

Selenium containing compounds, like 1,2,3-selenadiazoles are of increasing interest because of their chemical properties and biological applications such as anti-bacterial (El-Kashef *et al.*, 1986), anti-microbial (El-Bahaie *et al.*, 1990),anti-cancer (Plano *et al.*, 2010) and insecticidal (Padmavathi *et al.*, 2002) activities. It has been found that the introduction of 1,2,3-selenadiazole ring system increase the biological activity. Selenium atom has a much larger size and less electronegativity than the sulfur atom, selenium containig compounds influence the electro-optical properties of the small molecules (Velusamy *et al.*, 2005).

Glutathione peroxidases(GPx) are the antioxidant selenoenzymes protecting various organisms from oxidative stress by catalyzing the reduction of hydroperoxides at the expense of glutathione(GSH) (Stadtman, 1991). In view of the growing importance of selenium containing compounds, the crystal structure of the title compound has been carried out.

The *ORTEP* plot of the molecule is shown in Fig. 1. The bond lengths [Se1—N1] 1.870 (3) Å and [Se1—C1] 1.825 (4) Å are comparable with the values reported in the literature (Allen *et al.*, 1987). The selenadiazole ring is planar [with the maximum deviation for atom C2 of 0.004 (3)°]. Both the selenadiazole ring and chlorothiophene ring are lie in a common plane, the corresponding torsion angle is [C4—C3—C2—N2] 175.5 (3)°.

The packing of the molecules is shown in Fig. 2. The crystal packing is stabilized by C—H···N intermolecular interactions, linking the molecules to chains extending along the *b* axis.

### Experimental

A mixture of 2-acetyl-5-chlorothiophene (1 mmol), semicarbazide hydrochloride (2 mmol) and sodium acetate (3 mmol) in ethanol (10 ml) was refluxed for 4 hrs. After completion of the reaction as monitored by TLC, the mixture was poured into ice cold water and the resulting semicarbazone was filtered off. Then, a mixture of semicarbazone (1 mmol) and SeO_2_ (2 mmol) in tetrahydrofuran (10 ml) were refluxed on a water bath for 1 h. The selenium deposited on cooling was removed by filtration, and the filtrate was poured into crushed ice, extracted with dichloromethane, and purified by column chromatography using silica gel (60–120 mesh) with 97:3 petroleum ether: ethyl acetate as eluent to give 4-(5-chloro-2,5-dihydrothiophen-2-yl)-1,2,3-selenadiazole.

### Refinement

H atoms were positioned geometrically (C–H =0.93–0.97 Å) and allowed to ride on their parent atoms, with *U*_iso_(H) set to 1.2*U*_eq_(C).

### Crystal data

**Table:**

C_6_H_3_ClN_2_SSe	*F*(000) = 480
*M**_r_* = 249.57	*D*_x_ = 2.074 Mg m^−^^3^
Monoclinic, *P*2_1_/*c*	Mo *K*α radiation, λ = 0.71073 Å
Hall symbol: -P 2ybc	Cell parameters from 1558 reflections
*a* = 6.0412 (3) Å	θ = 2.1–28.3°
*b* = 19.5870 (11) Å	µ = 5.22 mm^−^^1^
*c* = 7.2010 (4) Å	*T* = 293 K
β = 110.257 (3)°	Black, white crystalline
*V* = 799.38 (7) Å^3^	0.22 × 0.20 × 0.18 mm
*Z* = 4	

### Data collection

**Table:**

Bruker SMART APEX CCD detector diffractometer	1978 independent reflections
Radiation source: fine-focus sealed tube	1558 reflections with *I* > 2σ(*I*)
Graphite monochromator	*R*_int_ = 0.044
ω scans	θ_max_ = 28.3°, θ_min_ = 2.1°
Absorption correction: multi-scan (*SADABS*; Bruker, 2008)	*h* = −8→6
*T*_min_ = 0.330, *T*_max_ = 0.391	*k* = −25→26
7064 measured reflections	*l* = −9→9

### Refinement

**Table:**

Refinement on *F*^2^	Primary atom site location: structure-invariant direct methods
Least-squares matrix: full	Secondary atom site location: difference Fourier map
*R*[*F*^2^ > 2σ(*F*^2^)] = 0.039	Hydrogen site location: inferred from neighbouring sites
*wR*(*F*^2^) = 0.121	H-atom parameters constrained
*S* = 1.01	*w* = 1/[σ^2^(*F*_o_^2^) + (0.0716*P*)^2^ + 0.3075*P*] where *P* = (*F*_o_^2^ + 2*F*_c_^2^)/3
1978 reflections	(Δ/σ)_max_ = 0.001
100 parameters	Δρ_max_ = 0.44 e Å^−^^3^
0 restraints	Δρ_min_ = −0.52 e Å^−^^3^

### Special details

**Table:**

Geometry. All e.s.d.'s (except the e.s.d. in the dihedral angle between two l.s. planes) are estimated using the full covariance matrix. The cell e.s.d.'s are taken into account individually in the estimation of e.s.d.'s in distances, angles and torsion angles; correlations between e.s.d.'s in cell parameters are only used when they are defined by crystal symmetry. An approximate (isotropic) treatment of cell e.s.d.'s is used for estimating e.s.d.'s involving l.s. planes.
Refinement. Refinement of *F*^2^ against ALL reflections. The weighted *R*-factor *wR* and goodness of fit *S* are based on *F*^2^, conventional *R*-factors *R* are based on *F*, with *F* set to zero for negative *F*^2^. The threshold expression of *F*^2^ > σ(*F*^2^) is used only for calculating *R*-factors(gt) *etc*. and is not relevant to the choice of reflections for refinement. *R*-factors based on *F*^2^ are statistically about twice as large as those based on *F*, and *R*- factors based on ALL data will be even larger.

### Fractional atomic coordinates and isotropic or equivalent isotropic displacement parameters (Å^2^)

**Table:**

	*x*	*y*	*z*	*U*_iso_*/*U*_eq_	
C1	0.3771 (6)	0.60797 (19)	0.2132 (5)	0.0397 (7)	
H1	0.5288	0.6078	0.2081	0.048*	
C2	0.2578 (6)	0.55180 (18)	0.2343 (4)	0.0333 (7)	
C3	0.3344 (6)	0.48114 (17)	0.2494 (5)	0.0328 (7)	
C4	0.5358 (6)	0.45391 (19)	0.2353 (5)	0.0412 (8)	
H4	0.6532	0.4799	0.2134	0.049*	
C5	0.5478 (6)	0.38256 (19)	0.2573 (5)	0.0413 (8)	
H5	0.6726	0.3562	0.2502	0.050*	
C6	0.3579 (6)	0.35688 (18)	0.2896 (5)	0.0376 (7)	
Cl1	0.29911 (17)	0.27326 (5)	0.32523 (15)	0.0529 (3)	
N1	−0.0347 (5)	0.62617 (17)	0.2239 (5)	0.0482 (8)	
N2	0.0334 (5)	0.56419 (16)	0.2406 (4)	0.0428 (7)	
S1	0.15880 (15)	0.41890 (5)	0.29606 (14)	0.0408 (2)	
Se1	0.19762 (7)	0.684213 (19)	0.19625 (6)	0.04757 (18)	

### Atomic displacement parameters (Å^2^)

**Table:**

	*U*^11^	*U*^22^	*U*^33^	*U*^12^	*U*^13^	*U*^23^
C1	0.0304 (16)	0.0409 (19)	0.0469 (18)	0.0004 (14)	0.0120 (14)	−0.0018 (15)
C2	0.0271 (15)	0.0379 (18)	0.0349 (15)	−0.0008 (12)	0.0107 (12)	−0.0014 (13)
C3	0.0286 (15)	0.0333 (16)	0.0368 (15)	−0.0044 (13)	0.0120 (13)	−0.0002 (13)
C4	0.0325 (17)	0.0404 (19)	0.056 (2)	−0.0015 (14)	0.0216 (15)	0.0038 (16)
C5	0.0336 (17)	0.042 (2)	0.055 (2)	0.0011 (14)	0.0232 (16)	−0.0024 (16)
C6	0.0353 (17)	0.0351 (18)	0.0427 (17)	−0.0030 (14)	0.0138 (14)	−0.0027 (14)
Cl1	0.0501 (6)	0.0375 (5)	0.0727 (6)	−0.0066 (4)	0.0234 (5)	0.0031 (4)
N1	0.0335 (15)	0.0461 (19)	0.068 (2)	0.0029 (13)	0.0221 (15)	0.0005 (15)
N2	0.0308 (14)	0.0443 (18)	0.0577 (18)	−0.0008 (13)	0.0208 (13)	−0.0007 (14)
S1	0.0289 (4)	0.0409 (5)	0.0559 (5)	−0.0041 (3)	0.0190 (4)	0.0004 (4)
Se1	0.0375 (2)	0.0371 (3)	0.0659 (3)	0.00047 (14)	0.01514 (19)	0.00089 (16)

### Geometric parameters (Å, º)

**Table:**

C1—C2	1.352 (5)	C4—H4	0.9300
C1—Se1	1.825 (4)	C5—C6	1.345 (5)
C1—H1	0.9300	C5—H5	0.9300
C2—N2	1.394 (4)	C6—Cl1	1.714 (4)
C2—C3	1.451 (5)	C6—S1	1.721 (4)
C3—C4	1.364 (5)	N1—N2	1.274 (4)
C3—S1	1.724 (3)	N1—Se1	1.870 (3)
C4—C5	1.406 (5)		
			
C2—C1—Se1	110.2 (3)	C5—C4—H4	123.4
C2—C1—H1	124.9	C6—C5—C4	112.2 (3)
Se1—C1—H1	124.9	C6—C5—H5	123.9
C1—C2—N2	115.1 (3)	C4—C5—H5	123.9
C1—C2—C3	128.0 (3)	C5—C6—Cl1	128.1 (3)
N2—C2—C3	116.9 (3)	C5—C6—S1	112.7 (3)
C4—C3—C2	129.5 (3)	Cl1—C6—S1	119.2 (2)
C4—C3—S1	111.3 (3)	N2—N1—Se1	111.1 (2)
C2—C3—S1	119.2 (2)	N1—N2—C2	116.7 (3)
C3—C4—C5	113.1 (3)	C3—S1—C6	90.63 (17)
C3—C4—H4	123.4	C1—Se1—N1	86.89 (15)
			
Se1—C1—C2—N2	−0.7 (4)	C4—C5—C6—S1	−0.4 (4)
Se1—C1—C2—C3	178.8 (3)	Se1—N1—N2—C2	−0.3 (4)
C1—C2—C3—C4	−4.0 (6)	C1—C2—N2—N1	0.7 (5)
N2—C2—C3—C4	175.4 (3)	C3—C2—N2—N1	−178.8 (3)
C1—C2—C3—S1	174.7 (3)	C4—C3—S1—C6	−1.4 (3)
N2—C2—C3—S1	−5.8 (4)	C2—C3—S1—C6	179.7 (3)
C2—C3—C4—C5	−179.7 (3)	C5—C6—S1—C3	1.0 (3)
S1—C3—C4—C5	1.4 (4)	Cl1—C6—S1—C3	−179.5 (2)
C3—C4—C5—C6	−0.7 (5)	C2—C1—Se1—N1	0.4 (3)
C4—C5—C6—Cl1	−179.8 (3)	N2—N1—Se1—C1	−0.1 (3)

### Hydrogen-bond geometry (Å, º)

**Table:**

*D*—H···*A*	*D*—H	H···*A*	*D*···*A*	*D*—H···*A*
C1—H1···N1^i^	0.93	2.62	3.545 (5)	171

Symmetry code: (i) *x*+1, *y*, *z*.
